# Plasma complement and vascular complement deposition in patients with coronary artery disease with and without inflammatory rheumatic diseases

**DOI:** 10.1371/journal.pone.0174577

**Published:** 2017-03-31

**Authors:** Kelly J. Shields, Tom Eirik Mollnes, Jon Roger Eidet, Knut Mikkelsen, Sven M. Almdahl, Barbara Bottazzi, Torstein Lyberg, Susan Manzi, Joseph M. Ahearn, Ivana Hollan

**Affiliations:** 1 Lupus Center of Excellence, Autoimmunity Institute, Department of Medicine, Allegheny Health Network, Pittsburgh, Pennsylvania, United States of America; 2 Department of Immunology, Oslo University Hospital, and K.G. Jebsen IRC, University of Oslo, Oslo, Norway; 3 Research Laboratory, Nordland Hospital, Bodø, and Faculty of Health Sciences, K.G. Jebsen TREC, University of Tromsø, Tromsø, Norway; 4 Centre of Molecular Inflammation Research, Norwegian University of Science and Technology, Trondheim, Norway; 5 Department of Medical Biochemistry, Oslo University Hospital, Oslo, Norway; 6 Department of Rheumatology, Lillehammer Hospital for Rheumatic Diseases, Lillehammer, Norway; 7 Department of Cardiothoracic and Vascular Surgery, University Hospital of North Norway, Tromsø, Norway; 8 Humanitas Clinical and Research Center, Rozzano, Italy; 9 Research Department, Innlandet Hospital Trust, Brumunddal, Norway; 10 Division of Rheumatology, Immunology and Allergy, Brigham and Women’s Hospital, Boston, Massachusetts, United States of America; 11 School of Medicine, Harvard University, Boston, Massachusetts, United States of America; Peking University First Hospital, CHINA

## Abstract

**Purpose:**

Inflammatory rheumatic diseases (IRD) are associated with accelerated coronary artery disease (CAD), which may result from both systemic and vascular wall inflammation. There are indications that complement may be involved in the pathogenesis of CAD in Systemic Lupus Erythematosus (SLE) and Rheumatoid Arthritis (RA). This study aimed to evaluate the associations between circulating complement and complement activation products with mononuclear cell infiltrates (MCI, surrogate marker of vascular inflammation) in the aortic media and adventitia in IRD_CAD_ and non-IRD_CAD_ patients undergoing coronary artery bypass grafting (CABG). Furthermore, we compared complement activation product deposition patterns in rare aorta adventitial and medial biopsies from SLE, RA and non-IRD patients.

**Methods:**

We examined plasma C3 (p-C3) and terminal complement complexes (p-TCC) in 28 IRD_CAD_ (SLE = 3; RA = 25), 52 non-IRD_CAD_ patients, and 32 IRD_No CAD_ (RA = 32) from the Feiring Heart Biopsy Study. Aortic biopsies taken from the CAD only patients during CABG were previously evaluated for adventitial MCIs. The rare aortic biopsies from 3 SLE, 3 RA and 3 non-IRD_CAD_ were assessed for the presence of C3 and C3d using immunohistochemistry.

**Results:**

IRD_CAD_ patients had higher p-TCC than non-IRD_CAD_ or IRD_No CAD_ patients (p<0.0001), but a similar p-C3 level (p = 0.42). Circulating C3 was associated with IRD duration (ρ, p-value: 0.46, 0.03). In multiple logistic regression analysis, IRD remained significantly related to the presence and size of MCI (p<0.05). C3 was present in all tissue samples. C3d was detected in the media of all patients and only in the adventitia of IRD patients (diffuse in all SLE and focal in one RA).

**Conclusion:**

The independent association of IRD status with MCI and the observed C3d deposition supports the unique relationship between rheumatic disease, and, in particular, SLE with the complement system. Exaggerated systemic and vascular complement activation may accelerate CVD, serve as a CVD biomarker, and represent a target for new therapies.

## Introduction

Inflammatory rheumatic diseases (IRD), such as systemic lupus erythematosus (SLE) or rheumatoid arthritis (RA), are associated with increased cardiovascular (CV) risk, which is not fully explained by traditional CV factors. The progression of CVD is in part related to an inflammatory response mediated by immune processes.

It is notable that not only systemic inflammation, but also inflammation within the vessel wall, including the deep vascular and perivascular layers, may play an essential role in atheroma formation and destabilization. Previous results from the Feiring Heart Biopsy Study (FHBS) demonstrated that mononuclear cell infiltrates (MCI) occur in the aortic media and adventitia of patients with coronary artery disease (CAD), with a higher occurrence and a greater extent in patients with IRD than non-IRD patients.[1, 2] It is possible that the sub-intimal inflammation may play a role in the pathogenesis of CVD, and premature CVD in IRD.

Circulating complement protein C3 (plasma C3, p-C3), a critical protein in the classical, mannose and alternative pathways, is a biomarker routinely used to monitor disease activity in SLE. Furthermore, C3 is known to be associated with subclinical measures of CVD such as vascular stiffness.[3–6] In animal models, vascular complement deposition associated with vascular stiffness is likely due to complement binding directly to the elastin and collagen fibers within the vascular wall.[7, 8] The potentially pathogenic role of complement in the development of CVD is highlighted in a C4-deficient patient with well-preserved elastic arteries despite aging and a medical condition associated with vascular stiffness.[9]

Complement activation products and, in particular, cell bound complement activation products (CB-CAPs) on circulating erythrocytes, platelets and lymphocytes, have been shown to be highly specific for SLE as diagnostic, monitoring and prognostic biomarkers.[10–12] Additional circulating biomarkers indicative of complement cascade activation and inflammation include the culminating formation of terminal complement complexes (p-TCC)[13] and circulating molecules such as pentraxin 3 (PTX3), which mediates the effects of complement activation within the alternative pathway and has been implicated in vascular inflammation.[13–15]

Exaggerated complement activation as a result of chronic inflammatory rheumatic disease (IRD) along with advanced CVD may increase both systemic and local inflammation causing vascular tissue damage, which may be involved in CVD pathogenesis. Therefore, in this study, we assessed expression of the parent complement factor C3 and its activation product C3d, along with the occurrence and extent of MCI (a marker of vascular inflammation), within the sub-intimal layers of the aortic wall in CAD patients with IRD (RA or SLE) or without IRD. Furthermore, we compared circulating levels of C3 and TCC within these patient groups, and examined associations with traditional CVD risk factors and other circulating inflammatory markers.

## Materials and methods

### Patients

Among patients undergoing coronary artery bypass grafting (CABG) included in FHBS, described elsewhere[1], we examined all RA patients fulfilling ACR 1987 criteria (RA, n = 25) and SLE patients fulfilling ARA 1982 criteria (SLE, n = 3) representing IRD_CAD_ (n = 28), and 52 non-IRD_CAD_ patients in whom aortic biopsies and/or blood samples were available. The exclusion criteria for both groups were age <18 years, psoriasis, and clinically significant infection or malignancy. The Regional Committee for Medical and Health Research Ethics approved the study protocol and all of the patients gave written informed consent.[1] Furthermore, we examined 32 RA patients fulfilling ACR criteria without CAD (IRD_No CAD_). The inclusion criteria for this IRD_No CAD_ group were age > 18 years, RA according to 1987 ACR criteria, absence of CAD (based on medical history and clinical and electrocardiographic findings) and any clinically significant infection or malignancy. The IRD_CAD_ and non-IRD_CAD_ groups were matched for age and sex. It was not feasible to identify and include older IRD patients without subclinical or clinical CAD manifestations. The IRD_No CAD_ group was matched for sex to the RA subgroup within IRD_CAD_.

### Data collection, aortic biopsies and laboratory analyses

The patients were examined by preoperative blood tests, interviews, physical examinations, and self-reported questionnaires, and by biopsies taken during CABG.[1]

#### Aortic biopsy

The details of the aortic biopsy have been previously described.[1] Briefly, aortic specimens were obtained from tissue routinely removed during CABG surgery in connection to establishment of aorto-coronary anastomoses. The aortic biopsies were obtained in a two-step process: at first, a part of the adventitia (5–10 mm) was removed from the ventral part of the ascending aorta. After that, an opening (4.8 mm in diameter) through the rest of the vessel wall in the same area was made using a punch device. The punch specimens contained a cross-section of intima, media and at times, a thin layer of adventitia. For surgical safety (to avoid thromboembolism), the anastomoses were preferably made at sites with less pronounced gross signs of atherosclerosis. Thus, the specimens were taken from relatively healthy areas of the ascendant aorta. Both types of specimens (the adventitia and the punch specimen) were fixed in formalin and embedded in paraffin [1].

#### Assessment of pathologic features

Previously, 3-μm thick sections, stained by hematoxylin-eosin (H&E), were examined in random order by light microscopy, by 2 independent pathologists blinded to the clinical data, for the presence and localization of atherosclerotic lesions and MCIs.[1] At the level where the biopsies are taken, the aortic adventitia is covered by the aortic part of the epicardium. MCIs adjacent to the mesothelium were classified as submesothelial (epicardial), unless there was diffuse spreading into the inner adventitia. The size of the largest MCI in a single section was classified according to the number of mononuclear cells: either none, <50 or ≥ 50 cells. Data on aortic MCI in the whole FHBS population (including several rheumatic diagnoses than the current sub-study) have been published previously [1].

#### Immunohistochemistry (IHC)

Serial sections of RA (n = 3), SLE (n = 3) and non-IRD (n = 3) were sent for an explorative IHC evaluation to the Lupus Center of Excellence in Pittsburgh. The rarity of the IRD patient biopsies, and, in particular the SLE biopsies, is a limitation within this IHC analysis; however, Aubry, et al, also performed a preliminary IHC analysis on limited RA coronary artery biopsies.[16]

Briefly, paraffin embedded samples were sectioned at 5μm. Standard IHC techniques were used to evaluate anti-human monoclonal C3 (Santa Cruz Biotechnology, Inc, Dallas, TX) and anti-human monoclonal C3d (Quidel Corporation, San Diego, CA) deposition within the tissue samples. Briefly, the tissue slides were deparaffinized using serial ethanol dilutions and rehydrated. Antigen retrieval was achieved through subsequent washes in 3.0% hydrogen peroxide, Tris-Buffered Saline and Tween 20 (TBST), microwave heated citrate, and then incubation in proteinase K with a rinse in TBST. Samples were then incubated in 3.0%BSA overnight at 4°C. HRP and AEC (DAKO kit) with a hematoxylin counterstain were completed the following day.

#### Blood tests

We collected fasting blood samples, and performed hematological, serological and plasma analyses as described previously.[1, 17] Except for routine tests, the samples were analyzed in batches (after storage in -80°C) in random order, by assessors blinded for the clinical data. Plasma was carefully obtained by rapid centrifugation and immediately stored at -80°C.[18] Circulating C3 was measured by nephelometry (Siemens BN ProSpec, Germany) and circulating TCC and pentraxin 3 levels were measured by commercially available ELISAs as previously described.[18, 19]

#### Statistical analysis

The chi-square test and Fisher’s exact test (for categorical variables), and the Kruskal-Wallis Test (for continuous skewed variables) were used to identify differences between the three groups (IRD_No CAD_ (RA only, Group 1), Non-IRD_CAD_ (Group 2), and IRD_CAD_ (SLE and RA, Group 3)). Post hoc pairwise comparisons were completed using either false discovery rate (categorical variables) or rank based nonparametric ANOVA (continuous variables) with adjusted p-values. Combining the two rheumatic diseases into one CAD group is not without precedence due to the rarity of SLE patients undergoing CABG.[20] Non-parametric Spearman correlations were performed between circulating complement factors (C3 and TCC) and known CVD risk factors and known circulating inflammatory markers only within the Non-IRD CAD and IRD (SLE and RA) CAD groups. No tissue samples could be obtained to evaluate the presence of MCIs in the IRD (RA only) no CAD group, therefore only the Non-IRD CAD and IRD CAD groups were evaluated using regression analyses. Simple logistic regression analysis was used to assess the relationships between the presence and size of adventitial MCIs (MCI ≥50 cells: yes/no) and a set of covariates, which included the complement proteins of interest and known CVD risk factors and circulating inflammatory markers. In multiple logistic regression analysis, we created 3 separate models adjusting for **1)** age and gender, **2)** age, gender, and traditional CVD risk factors (a. history of thrombosis (which included any myocardial infarction (MI), stroke, acute coronary syndrome (ACS), deep vein thrombosis (DVT), or pulmonary embolism (PE)), b.) hypertension, c.) current smoker, d.) family history of CVD, e.) BMI), **3)** age, gender, and circulating inflammatory markers (a.) C3, b.) TCC, c.) PTX3, d.) CRP). All estimates were performed with SAS for Windows, version 9.4 (SAS, Cary, NC) and R-Project, package fifer for post hoc pairwise comparison using false discovery rate. The level of significance was set at 0.05, and all statistical tests were 2-sided.

## Results

### Demographics and clinical characteristics

The characteristics of the patients are shown in Table 1. The median (25^th^-75^th^%) age of IRD_No CAD_ was 57(52–66), Non-IRD_CAD_ was 70.0 (64–78), and IRD_CAD_ was 70.5 (62–76)years old. Twenty-four IRD_No CAD_ (75%), 18 non-IRD_CAD_ (35%), and 13 IRD_CAD_ (46%: SLE, n = 2 and RA, n = 11) patientswere female (Table 1). All participants were from Norway, and all were Caucasian. (S1 Table)

**Table 1:** Participant demographics, clinical circulating panels, and CVD history.

	Group 1 IRD_No CAD_	Group 2 Non-IRD_CAD_	Group 3 IRD_CAD_ (SLE and RA)	Overall p-value	Post Hoc Pairwise Comparisons†
	n = 32	n = 52	n = 28		(Group 1 vs 2 vs 3)
**Gender, Female**	24/32(75%)	18/52(35%)	13/28(46%)	0.002	1 v 2; 1 v 3
**Age, years**	57(52–66)	70(64–78)	70.5(62–76)	0.0001	1 v 2; 1 v 3
**BMI, kg/m^2^**	24(22–27)	25(24–28)	25(21–28)	0.52	--
**Smoke ever**	0/31(0%)	25/52(48%)	11/28(39%)	<0.0001	1 v 2; 1 v 3
**Smoke now**	18/31(58%)	7/52(13%)	6/28(21%)	<0.0001	1 v 2
**CRP, mg/L**	3(2–8)	2.3(1.1–5.1)	9.9(2.5–20)	0.0016	2 v 3
**Triglycerides**	1.15(0.79–1.4)	1.45(1–1.8)	1.75(1–2)	0.026	1 v 3
**Cholesterol**	5.45(4.6–6)	4.95(4.3–5.4)	4.8(4.3–5.6)	0.15	--
**LDL**	3.4(2.4–3.9)	2.9(2.6–3.5)	3(2.7–3.65)	0.92	--
**PTX3, ng/ml**	N/A	1.34(0.78–2.1)	1.83(1.2–2.9)	0.02	--
**ESR, mm/hour**	13(7–21)	13(6–23)	24(16–48)	0.005	1 v 3; 2 v 3
**MCV, fL/RBC**	89.5(86–94)	23(18–32)	18(15–25)	0.0001	1 v 2; 1 v 3
**TCC, CAU/ml**	0.7(0.4–0.8)	0.7(0.6–0.8)	1.3(0.9–1.6)	<0.0001	1 v 3; 2 v 3
**Circulating C3, g/L**	1.11(0.98–1.2)	1.15(1.0–1.4)	1.13(1.1–1.3)	0.42	--
***CVD History***					
**Hypertension**	7/28(25%)	30/52(58%)	18/28(64%)	0.005	1 v 2; 1 v 3
**Family History CVD**	9/27(33%)	32/52(81%)	19/28(68%)	<0.0001	1 v 2; 1 v 3
**Myocardial Infarction**	0/32(0%)	23/52(44%)	20/28(71%)	<0.0001	1 v 2; 1 v 3
**Acute Coronary Syndrome**	0/32(0%)	12/42(23%)	9/28(32%)	0.001	1 v 2; 1 v 3
**Stroke**	0/32(0%)	3/50(6%)	3/28(11%)	0.10	--
**DVT**	0/32(0%)	3/42(7.1%)	0/27(0%)	0.18	--
**CAD duration, months**	N/A	41(12–122)	24(6–113)	0.34	--
***Localized Aortic Inflammation***					
MCI*	N/A	10/50(20%)	13/25(52%)	0.005	--
MCI ≥50**	N/A	5/50(10%)	9/25(36%)	0.01	--

Median (25th-75th%) or n/total (%) Body mass index (BMI), C-reactive protein (CRP), Low Density Lipoprotein (LDL), Pentraxin3 (PTX3), Erythrocyte Sedimentation Rate (ESR), Mean Corpuscular Volume (MCV), Terminal Complement Complex (TCC, reference range (ref): <0.7 CAU/ml)[18], Complement C3 (ref, assay specific: 0.70–2.00 g/L), Deep Vein Thrombosis (DVT), Mononuclear cell infiltrates (MCI)

* presence within adventitial aorta,

**extent (≥50) within adventitial aorta.

Post hoc pairwise comparisons adjusted p-values: False Discovery Rate (FDR) (for categorical variables) and rank based nonparametric ANOVA (for continuous variables).

^†^Only the groups with statistically significant post hoc comparisons are shown.

As expected, the IRD_CAD_ patients had higher circulating levels of systemic inflammatory biomarkers (ESR, CRP and PTX3) when compared to the IRD_No CAD_ and Non-IRD_CAD_. Circulating TCC was significantly higher in IRD_CAD_ (SLE: 1.4(0.85–1.75) and RA: 1.3(0.9–1.55) CAU/ml) when compared to IRD_No CAD_ or non-IRD_CAD_; however, there was no difference between IRD_No CAD_ and non-IRD_CAD_. Furthermore, IRD_CAD_ also had a higher occurrence of previous myocardial infarctions (MIs) when compared to the non-IRD_CAD_ patients. (Table 1)

### Relationship between circulating C3 and TCC levels with clinical and laboratory variables

In non-IRD_CAD_ patients, circulating C3 levels were positively correlated to BMI (ρ, p-value: 0.51, 0.0001) and CRP (0.39, 0.004), and circulating TCC levels were positively correlated with CRP (0.35, 0.011). In the IRD_CAD_ group, circulating C3 levels were significantly associated only with IRD disease duration (0.46, 0.03).

### Relationship between MCI and clinical and laboratory variables

In simple logistic regression analysis, the presence of IRD was significantly associated with the presence of MCI (OR[95% CI]: 4.30[1.5–12]), and even more strongly with the presence of MCI ≥ 50 (5.06[1.5–17]), in the aortic adventitia. The presence of MCI was also positively related to CRP, and the occurrence of ≥ 50 MCI to hypertensive status and current smoking. (Table 2)

**Table 2:** Simple logistic regression analysis on presence and extent of adventitial MCI (≥50) with clinical and laboratory parameters.

	Presence of MCI	p-value	Extent of MCI > = 50	p-value
	(OR[95%CI])		(OR[95%CI])	
**Presence of IRD**	4.30[1.5–12]	0.006	5.06[1.5–17]	0.01
**Female**	0.79[0.28–2.2]	0.64	1.24[0.38–4.0]	0.72
**Age**	0.99[0.94–1.0]	0.54	0.99[0.94–1.0]	0.73
**BMI**	0.87[0.75–1.0]	0.07	0.91[0.77–1.1]	0.28
**C3**	1.90[0.28–13]	0.52	8.94[0.88–91]	0.064
**CRP**	1.11[1.0–1.2]	0.008	1.05[0.98–1.1]	0.13
**Cholesterol**	1.06[0.64–1.7]	0.83	1.12[0.64–2.0]	0.7
**LDL**	1.07[0.60–1.9]	0.82	1.11[0.59–2.1]	0.74
**Triglycerides**	1.10[0.53–2.3]	0.81	1.32[0.60–2.9]	0.49
**Pentraxin 3**	0.60[0.31–1.1]	0.12	0.927[0.46–1.9]	0.83
**MCV**	0.98[0.95–1.0]	0.23	0.985[0.95–1.0]	0.40
**TCC**	2.46[0.78–7.8]	0.13	2.42[0.66–8.9]	0.18
History of thrombosis*	1.11[0.38–3.2]	0.85	1.22[0.34–4.4]	0.76
**Hypertension**	2.62[0.89–7.7]	0.08	5.44[1.1–26]	0.04
**Smoke current**	1.79[0.50–6.4]	0.37	4.29[1.1–16]	0.034

Body mass index (BMI), C-reactive protein (CRP), Low Density Lipoprotein (LDL), Pentraxin3 (PTX3), Mean Corpuscular Volume (MCV), Terminal Complement Complex (TCC).

*History of Thrombosis: myocardial infarction (MI), stroke, acute coronary syndrome (ACS), deep vein thrombosis (DVT), or pulmonary embolism

In multiple logistic regression analysis, the association between IRD and the presence of adventitial MCIs and the presence of MCI ≥ 50 was significant in Model 1and 2. In model 3, adjusting for circulating inflammatory markers slightly attenuated the association between IRD and MCI (p = 0.055), but the association to MCI ≥ 50 remained statistically significant (p = 0.03).(Table 3)

**Table 3:** Multiple logistic regression analysis on presence and extent of adventitial MCI (≥50) with clinical and laboratory parameters.

**Adjusting for gender and age**					
**Model I**		**Presence of MCI**	**p-value**	**Presence of MCI ≥50**	**p-value**
	**Presence of SLE and RA**	4.68 [1.6–14]	0.005	5.01 [1.4–17]	0.01
	**+ Female**	0.633[0.20–2.0]	0.44	1.05[0.28–3.9]	0.94
	**+ Age**	0.99[0.94–1.0]	0.76	0.99[0.93–1.1]	0.79
**Adjusting for traditional CVD risk factors**					
**Model II**		**Presence of MCI**	**p-value**	**Presence of MCI ≥50**	**p-value**
	**Presence of SLE and RA**	5.66[1.5–21]	0.01	6.13[1.3–30]	0.03
	**+Female**	0.313[0.07–1.3]	0.11	0.822[0.16–4.3]	0.82
	**+Age**	0.971[0.91–1.0]	0.35	0.990[0.92–1.1]	0.80
	+History of Thrombosis*	0.654[0.17–2.5]	0.53	0.605[0.12–3.1]	0.55
	**+Hypertension**	3.53[0.97–13]	0.06	10.1[1.1–92]	0.04
	**+Smoke, current**	1.80[0.37–8.8]	0.47	8.40[1.3–55]	0.03
	**+Family History of CVD**	2.70[0.55–13]	0.22	3.12[0.46–21]	0.24
	**+BMI**	0.790[0.64–0.97]	0.026	0.893[0.70–1.1]	0.35
**Adjusting for circulating inflammatory markers**					
**Model III**		**Presence of MCI**	**p-value**	**Presence of MCI ≥50**	**p-value**
	**Presence of SLE and RA**	4.87[0.97–24]	0.055	6.78[1.2–40]	0.03
	**+Female**	0.659[0.16–2.7]	0.56	1.02[0.22–4.7]	0.98
	**+Age**	0.999[0.93–1.1]	0.98	1.01[0.94–1.1]	0.77
	**+CRP**	1.12[1.0–1.2]	0.03	1.01[0.91–1.1]	0.86
	**+C3**	1.13[0.10–13]	0.92	13.7[0.93–203]	0.06
	**+TCC**	0.941[0.16–5.7]	0.95	1.09[0.15–7.9]	0.93
	**+Pentraxin 3**	0.271[0.10–0.71]	0.008	0.563[0.22–1.4]	0.22

Body mass index (BMI), C-reactive protein (CRP), Low Density Lipoprotein (LDL), Pentraxin3 (PTX3), Terminal Complement Complex (TCC).

*History of Thrombosis: myocardial infarction (MI), stroke, acute coronary syndrome (ACS), deep vein thrombosis (DVT), or pulmonary embolism

### Immunohistochemistry: C3 and C3d detection

Parent complement protein C3 was detected in a diffuse pattern within the aortic media and adventitia of all samples, regardless of IRD status.(not shown)

**Fig 1:**
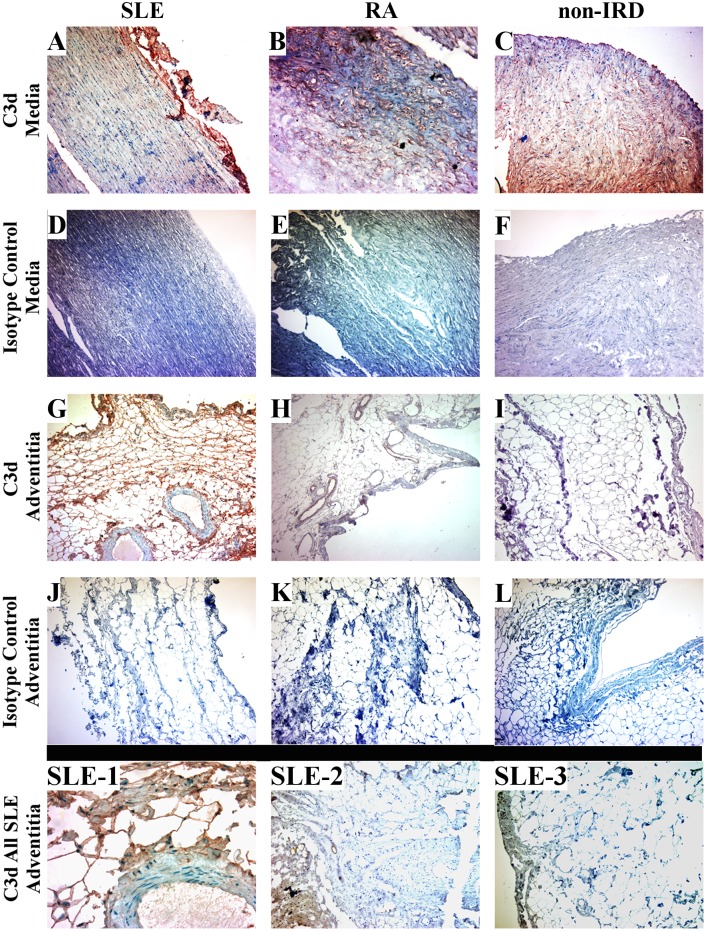
Complement activation product, C3d, presence within the vascular media and adventitia sections for IRD_CAD_ (SLE and RA) and non-IRD_CAD_ only patients. **A-C)** C3d deposition in the aorta media of all biopsies; **D-F)** Isotype control—aorta media; **G-I)** C3d deposition in the aorta adventitia: diffusely in SLE **(G)**, focally in RA **(H)**, and none in non-IRD **(I)**; **J-L)** Isotype control—aorta adventitia. All images 10x.

More importantly, the C3 activation product, C3d, was detected in a diffuse pattern throughout the aortic media of all tissue specimens regardless of IRD (Fig 1A–1C). However, C3d deposition was detected exclusively in all SLE adventitial samples (Fig 1G and SLE-1-3). One SLE adventitial sample had an overall diffuse pattern of C3d deposition (Fig 1G, SLE-1) while the other two SLE adventitia had a more localized pattern (Fig 1: SLE-2 and -3). Limited focal deposition surrounding the adventitial vasculature occurred also in 1 RA patient (Fig 1H). No C3d was detected in the non-IRD_CAD_ adventitial samples (Fig 1I).

**SLE adventitia only—different C3d deposition patterns: SLE-1)** Higher magnification (40x) of the diffuse C3d pattern found in the SLE biopsy (Fig 1G); **SLE-2 and -3)** Other 2 SLE adventitial biopsies with a localized C3d presence, (10x). C3d = red-brown for all images.

## Discussion

To our knowledge, this is the first study to evaluate complement protein deposition within the vessels of SLE, RA, and non-IRD patients with CAD. The findings support the unique relation between complement and IRD, in particular SLE. From these findings, two important questions arise, which include the source and the targets of the complement and the complement activation products.

Traditionally, the source of complement for deposition within various tissues types has been associated with systemic circulation. Another possible source is the perivascular adipose tissue (PVAT) surrounding the vascular wall, which is not anatomically separated from the adventitia. As mentioned above, complement activation in the adventitia occurred only in a subset of IRD_CAD_ patients. The adventitial samples have an extensive number of adipocytes that are not found in the media samples. Adipose tissue is known to produce complement proteins [21], and visceral adipose tissue is strongly associated with CVD. IRD patients are predisposed to greater volumes of visceral adipose when compared to the general population.[22, 23] We have previously found increased volumes of localized PVAT surrounding the aorta in women with SLE when compared to their age- and race-matched healthy controls despite no differences in BMI.[24] These small visceral vascular and perivascular depots may have a localized influence on vascular health and contribute to the progression of CVD from the ‘outside-in’.[25]

Liu et al has established that various circulating cell types are targets of complement activation products (cell bound-CAPs), which are highly specific to SLE and associated with all-cause mortality and stroke.[10–12] Additionally, in mouse models of CVD, we have found complement proteins (C3 and C4) targeting the collagen and elastin within the aortic vascular wall regardless of luminal athero-lesion development, and associated with increased vascular stiffness.[7,8] The role of complement contributing to vascular stiffness and CVD is highlighted by a remarkable clinical example of a 51 year old, kidney transplant candidate with hereditary complement protein C4 deficiency. The patient was found to have a central vascular stiffness similar to that of an adolescent and a carotid wall thickness better than normal for the patient’s age group.[9] Complement activation products may target both circulating immune cells and structural proteins of the vascular wall. Each of these mechanisms may be important biomarkers of CV events [12] and in CVD pathogenesis [6–9].

Our present findings show that parent complement deposition exists within the vascular wall in patients with CAD regardless of IRD status. However, those with SLE or RA, exhibit inflammation both within the vascular wall, marked by increased MCIs and complement activation products, and systemically, through higher circulating levels of inflammatory biomarkers. The younger RA group with no subclinical or clinical history of CAD had circulating TCC levels similar to that of nonIRD CAD patients approximately 15 years older and in need of CABG. These high circulating TCC levels support existing literature citing there is systemic, circulating inflammation occurring in IRD patients that may be contributing to CAD progression. The increased circulating TCC in the IRD_CAD_ (SLE and RA) group reflects increased basic complement activation [14], and may be mediated by increased levels of PTX3, which is known to regulate complement activation.[15]

Notably, PTX3 is an acute phase reactant and higher PTX3 levels were protective against the presence of adventitial MCI. A potential protective role of PTX3 in CVD has been demonstrated in previous studies. The protective role of PTX3 in CVD is supported by an animal model of acute MI, which showed increased myocardial damage along with increased C3 deposition in PTX3 deficient mice.[26] PTX3 is known to modulate activation of the alternative pathway through Factor H and down-regulate exaggerated inflammation by suppressing leucocyte extravasation through P-selectin.[27–29] Additionally, circulating C3 was positively correlated with IRD duration in our study thus underscoring the potential role of long-term IRD-associated inflammation on cardiovascular health.

Furthermore, despite no differences in age and the advanced CAD necessitating CABG for both groups, the SLE and RA CAD patients suffered a higher number of MIs compared to the non-IRD_CAD_ group, which highlights the accelerated CVD progression within IRD. Taken together, our results support the notion that controlling complement activation may be particularly crucial for both systemic and vascular inflammation in SLE and RA, in order to reduce CVD morbidity.

There are some limitations in this study including the relatively small SLE and RA population and the cross-sectional design. The rarity of this type of tissue is evidenced by the FHBS obtaining only 3 SLE CABG aortic medial and adventitial biopsies during a five-year period. The surgical biopsies have a great advantage compared to autopsy specimens as they are not deteriorated by post mortem processes. Additionally, we have not been able to examine other biopsied tissue. Due to ethical reasons and feasibility, we were not able to obtain aortic specimens from either IRD population without CAD or healthy individuals for comparison.

## Conclusions

In conclusion, among CABG patients, IRD patients had higher p-TCC and more complement activation in the vascular adventitia when compared to non-IRD patients. The pronounced adventitial complement activation in SLE patients might be due to the unique role of complement in SLE. Exaggerated systemic and vascular complement activation may accelerate CVD development, but also may serve as CVD biomarkers and represent new targets for CVD therapies.

## Supporting information

S1 Table Supporting cohort data. Clinical, demographic, and quantifiable data of the cohort.
(XLSX)
